# Inverse association between the molecular spreading of IgE to grass pollen and the IgE response to *Dermatophagoides pteronyssinus* among children with seasonal allergic rhinitis^☆^

**DOI:** 10.1016/j.waojou.2024.100975

**Published:** 2024-09-19

**Authors:** Giulia Brindisi, Francesca Cipriani, Ekaterina Potapova, Salvatore Tripodi, Valentina Panetta, Roberto Bernardini, Carlo Caffarelli, Antonella Casani, Rosa Cervone, Loredana Chini, Pasquale Comberiati, Giovanna De Castro, Michele Miraglia Del Giudice, Iride Dello Iacono, Andrea Di Rienzo Businco, Stephanie Dramburg, Marcella Gallucci, Arianna Giannetti, Viviana Moschese, Ifigenia Sfika, Elena Varin, Giampaolo Ricci, Gerald Reese, Anna Maria Zicari, Paolo Maria Matricardi

**Affiliations:** a Department of Mother-Child, Urological Science, La Sapienza University, Rome, Italy; b Primary Care Pediatrics, AUSL Bologna, Bologna, Italy; c Charité – Universitätsmedizin Berlin, Corporate Member of Freie Universität Berlin and Humboldt-Universität zu Berlin, Department of Pediatric Respiratory Medicine, Immunology and Critical Care Medicine, Augustenburger Platz 1, 13353 Berlin, Germany; d Pediatric Department and Pediatric Allergology Unit, Sandro Pertini Hospital, Rome, Italy; e Consultancy & Training, Biostatistics, L'altrastatistica srl, Rome, Italy; f Pediatric Unit, San Giuseppe Hospital, Empoli, Italy; g Clinica Pediatrica, Department of Medicine and Surgery, University of Parma, Parma, Italy; h Pediatra di Libera Scelta, Benevento, Italy; i UOSD di Immunopatologia ed Allergologia Pediatrica, Policlinico Tor Vergata, Università di Roma Tor Vergata, Rome, Italy; j Department of Clinical and Experimental Medicine, Section of Paediatrics, University of Pisa, Pisa, Italy; k Dipartimento della Donna, del Bambino e di Chirurgia Generale e Specialistica, Università della Campania Luigi Vanvitelli, Naples, Italy; l Pediatric Unit, Fatebenefratelli Hospital, Benevento, Italy; m Pediatric Unit, IRCCS Azienda Ospedaliero-Universitaria di Bologna, Bologna, Italy; n Pediatric Intermediate Care Unit, Fondazione IRCCS Ca’ Granda Ospedale Maggiore Policlinico, Milan, Italy; o State Institute of Health, Bavarian Health and Food Safety Authority, Erlangen, Germany

**Keywords:** Allergic rhinitis, Children, Dermatophagoides pteronyssinnus, *Phleum pratense*, Molecular spreading

## Abstract

**Background:**

Seasonal allergic rhinoconjunctivitis (SAR) is a worldwide health problem, especially in Westernized countries. Previous studies of the “Panallergens in Pediatrics” (PAN-PED) cohort found that molecular spreading (ie, the progressive increase in serum specific IgE antibody levels) of the IgE response to the grass pollen, *Phleum pratense*, molecules is directly associated with polysensitization to pollen in general.

The research question is aimed at verifying whether this association can also be detected for non-pollen allergens, specifically *Dermatophagoides pteronyssinnus* (*D.pt*), to better understand the relationship between a perennial allergen (*D.pt*) and a seasonal allergen (*Phleum pratense*).

To this end, our first objective was to analyze the biobank of the PAN-PED cohort serum by measuring the IgE levels to *D.pt* and its major recombinant molecules (Der p1, Der p2, Der p23); subsequently we investigated their correlation towards *Phleum pratense* IgE response, studying also the relationship between the molecular spreading of these 2 different allergens.

**Methods:**

Among 1120 patients positive to *Phleum pratense*, 638 were also sensitized to *D.pt*. Patients underwent skin prick tests (SPT) for inhalant extracts, and their serum was tested for total IgE (tIgE), and sIgE to pollen and perennial allergens. Considering the molecular allergen detection through the component resolved diagnosis (CRD), out of 638 patients, 146 were further investigated by performing IgE tests of the 3 major D.pt. molecules: Der p1, Der p2, and Der p23.

**Results:**

We found that a broader molecular response to *Phleum pratense* molecules, assessed by CRD, was associated with higher tIgE levels, polysensitization to pollens, and higher IgE levels to pollens, but also to lower IgE levels to *D.pt* and lower degree of sensitization to rDer p1, r Der p2, and rDer p23. In a multivariate linear model, the number of *Phleum pratense* molecules recognized by IgE was still inversely associated with the IgE level to *D.pt* extract.

**Conclusions:**

The main finding of this study was the detection of an inverse association, never described in the literature, between the molecular spreading of the IgE response to *Phleum pratense* and the IgE response to *D.pt*. This led us to speculate on the etiopathogenetic hypothesis according to which, among the majority of pollen allergic patients, a strong and molecularly diversified IgE response may be limited to pollen allergens and may be preventing or contrasting the development of an equally strong and diversified IgE sensitization to *D.pt* molecules. The biological mechanisms underlying this phenomenon deserve to be investigated.

**KEY MESSAGE**

In this study we investigated the presence of an inverse association between the molecular spreading of IgE to *Phleum Pratense* and the IgE response to *Dermatophagoides pteronyssinus* among children with seasonal allergic rhinitis evaluated among the large cohort of “Panallergens in Pediatrics” patients.

A strong and molecularly diversified IgE response may be limited to pollen allergens, preventing or contrasting the development of an equally strong and diversified IgE sensitization to *Dermatophagoides pteronyssinus* molecules. However, the biological mechanisms underlying this phenomenon need to be further investigated and deserve attentionn

## Introduction

Allergic rhinitis (AR) is one of the most common allergic diseases affecting millions of people worldwide, with a steadily increasing prevalence in Western countries,^1^ associated with asthma in up to 40% of cases due to continuity between the upper and lower airways.^2^, ^3^, ^4^, ^5^, ^6^

In children, AR symptoms affect the quality of life of patients and their parents with a high socio-economical burden due to parents’ absence from work, drug costs, and emergency room visits.^6^, ^7^, ^8^, ^9^

A recent study conducted in Italy showed that the prevalence of sensitization to grass pollens varies according to the diversity of regional climates, with the highest prevalence in the center of Italy (54%).^10^ Instead, another countrywide Italian study of children with seasonal allergic rhinitis (SAR) showed the prevalence of IgE sensitization to *Phleum pratense* has the highest percentage in northern regions (95.1%).^7^

Considering allergy molecular diagnostics,^11^^,^^12^ it has shown that IgE sensitization to *Phleum pratense* often begins pre-clinically—years before the onset of SAR as a weak, mono- or oligo-molecular profile (2–4 molecules), which in many patients evolves rapidly to a strong, poly-molecular sensitization (5–8 molecules) stage.^8^^,^^13^^,^^14^ IgEs to Phl p1, the most important initiator of *Phleum pratense* sensitization,^8^^,^^13^ appears up to 5 years before any allergic symptoms.^8^ According to a frequent scenario defined as “molecular spreading”,^8^^,^^11^^,^^13^ the broadening sensitization process continues with other *Pheum pratense* moleculas, such as Phl p 5 and/or Phl p 4, followed by Phl p 2 and/or Phl p 6 and/or Phl p 11, and in a few cases by Phl p 12 and Phl p 7.^8^^,^^12^^,^^15^, ^16^, ^17^, ^18^

Parental hay fever and the family context are pivotal factors influencing the occurrence of the long-term evolution of the IgE responses to grass pollen, promoting the molecular spreading and facilitating the progression of sensitization to multiple allergenic sources.^11^^,^^19^, ^20^, ^21^, ^22^, ^23^, ^24^, ^25^, ^26^ Accordingly, in a large population of children with SAR participating in the “Panallergens in Pediatrics” (PAN-PED) study, we found that the number of allergenic molecules of *Phleum pratense* recognized by serum IgE is directly associated with the level of total and grass pollen-specific IgE, and with IgE sensitization to other pollen.^11^ In contrast, we also unexpectedly found that the number of sensitizations to individual molecules of *Phleum pratense* extract was inversely correlated with the level of IgE antibodies to an allergenic extract of *Dermatophagoides pteronyssinus* (*D.pt*).^11^

Therefore, the present study aimed to further investigate this surprising and fortuitous discovery by analyzing and characterizing the type of relationship between allergens so different from each other, on the one hand a seasonal allergen, such as *Pleum pratense*, and on the other hand a perennial one, such as *D.pt*.

To this end, we further tested, through the allergy molecular diagnostic, the occurrence and levels of IgE to Der p 1, Der p 2, and Der p 23 in the same population to test the hypothesis that the relationship of the molecular spreading of the IgE responses to *Phleum pratense* is – in our population sample – inversely related to the molecular spreading of the IgE response to *D.pt.*

## Materials and methods

### Study populations

The “Panallargen in Pediatrics” (PAN-PED) enrolled 1360 patients during routine appointments in 16 pediatric outpatient clinics in 14 Italian cities between 2009 and 2011.^7^ Criteria for eligibility were as follows: (i) age 4–18 y; (ii) history of pollen-induced allergic rhinitis (AR) and/or allergic asthma in one of the 2 last pollen seasons; and (iii) positive skin prick tests (SPTs) for the relevant pollen extracts. Exclusion criteria were as follows: (i) previous specific allergen immunotherapy (AIT) for any pollen allergen; and (ii) any other severe chronic disease. Recruited children's parents answered a standardized questionnaire, and patients underwent SPTs and a blood draw.

### Skin prick tests

SPTs were performed using a standard panel of commercial extracts (ALK-Abello, Milan, Italy) of seasonal airborne allergens (*Phleum pratense, Cynodon dactylon,* pellitory*, Plantago lanceolata, Chenopodium album,* mugwort*, Salsola kali,* ragweed, cypress, birch, plane tree*, Olea europaea,* elm, hazel*),* food allergens *(peach, apple, wheat, soybean, peanut, hazelnut*), and latex. A commercial date palm pollen extract enriched in profilin (ALK-Abello, Milan, Italy) was used to detect profilin sensitization. Histamine 0.1 mg/ml and glycerol solution were positive and negative controls, respectively. Morrow-Brown needles were used to prick the skin, and the wheal reactions were read after 15 min. A wheel ≥3 mm after subtracting the negative control was regarded as positive.

### IgE to extracts and molecules; tIgE

Serum samples were tested for tIgE and sIgE to pollen extracts (*Cynodon dactylon*, *Betula verrucosa*, *Cupressus arizonica*, *Platanus orientalis*, *Olea europaea*, *Parietaria judaica*, *Artemisia vulgaris*, *Plantago lanceolata*, and *Ambrosia artemisiifolia*) and to perennial airborne allergens (*Dermatophagoides pteronyssinus*, cat dander and *Alternaria alternata*) (ImmunoCAP FEIA; Phadia, Uppsala, Sweden). IgE to a panel of Phleum pratense molecules (rPhl p 1, rPhl p 2, nPhl p 4, rPhl p 5b, rPhl p 6, rPhl p 7, rPhl p 11, rPhl p 12) and to a panel of Dermatophagoides pteronyssinus molecules (rDer p 1, rDer p 2, rDer p 23) (ImmunoCAP FEIA) was measured in sera of patients showing a SPT wheal reaction ≥3 mm elicited by the *Phleum pretense* extract or with IgE to the extract of *Dermatophagoides pteronyssinus*, respectively. Results were considered positive if > 0.35 kUA/L.

### Statistics

Data were summarized as numbers (n) and frequencies (%) if they were categorical and as mean and standard deviation (SD) if quantitative. The average concentration of tIgE and sIgE levels were calculated as geometric mean, and only the positive serum samples were considered for sIgE antibodies (see the above definition). The prevalences of IgE sensitization (≥0.35 kUA/L) to the 8 *Phleum pratense* molecules were examined. Linear regression using log transformed IgE was used to calculate the trend on Number of Molecules, respectively, on total and serum IgE levels, as well as on *D.pt* prevalence. Univariable and multivariable logistic regression was used to estimate the effect of the number of *Phleum pratense* molecules recognized by IgE on patients with positive sIgE to *Dermatophagoides pteronyssinus*. We excluded patients with sporadic missing values in IgE tests (due to insufficient serum volume). A p value of <0.05 was considered statistically significant. Statistical analysis was performed by using Stata 16.1.

## Results

### Population sample

**Fig. 1:**
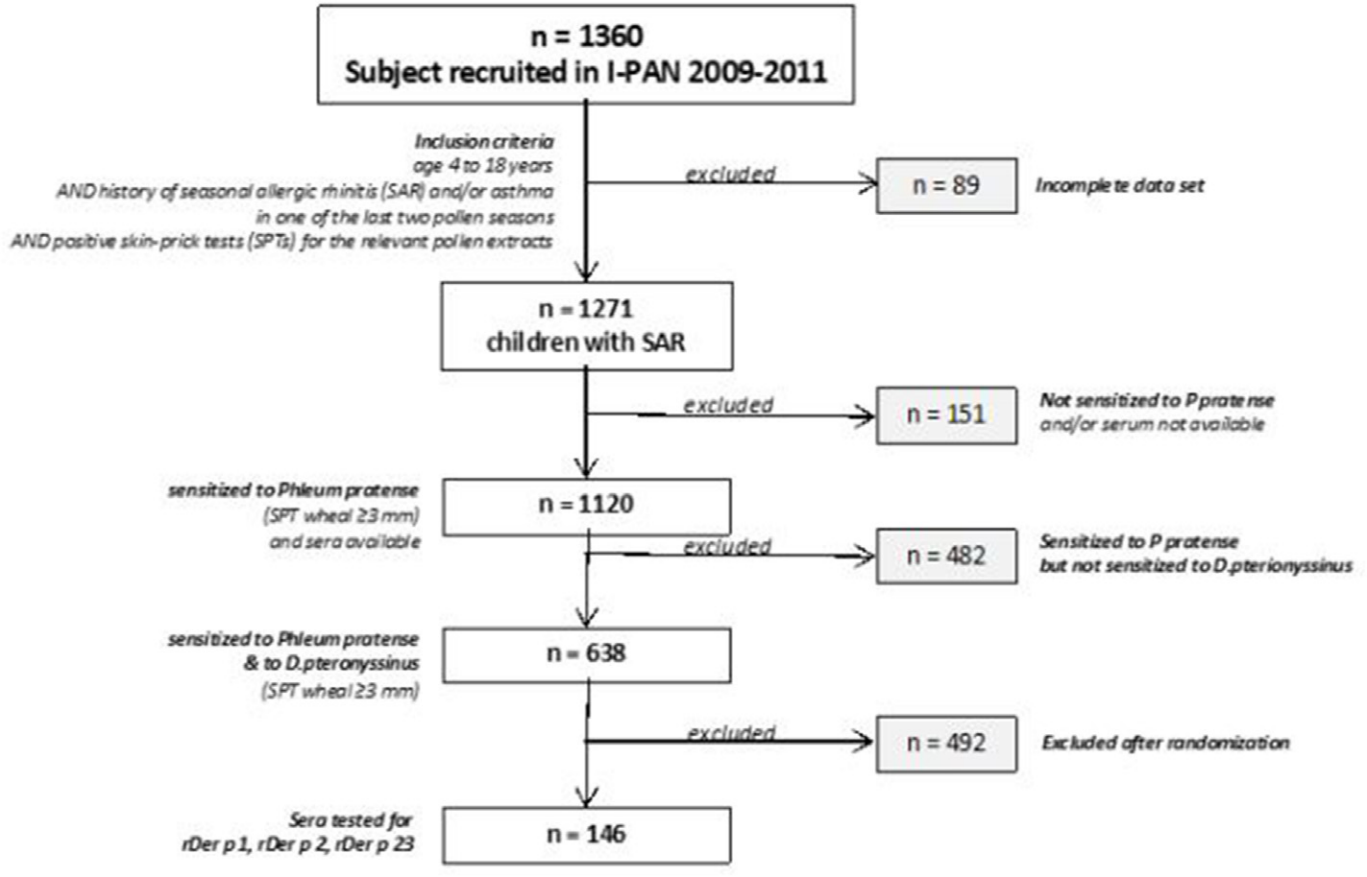
The “Panallergens in Pediatrics” study population: flow chart with inclusion and exclusion criteria in the analysis of grass pollen allergic paients with or without IgE sensitization to house dust mites. ***D.pt*:** Dermatophagoides pteronyssinus **Phl p1**: Phleum pretense, **IgE:** Immunoglobulin IgE, **Der p1, p2, p23:** Dermatophagoides pteronyssinus.

Among the 1360 patients of the study, 1271 could be serologically tested, 1120 were sentitized to grass pollen and included in the present analysis [Fig. 1]. Among them, 638 patients were also sensitized to *D.pt*. Of the 638 patients, 146 were more deeply investigated, by performing IgE tests to the 3 major recombinant allergenic proteins Der p 1, Der p 2, and Der p23. The main characteristics of patients included or excluded at different steps of the study were reported in Table 1. Patients sensitized to *D.pt* showed longer duration of SAR, higher number of winter months with symptoms, and higher rate of comorbid asthma compared to patients sensitized to grass pollen but not sensitized to *D.pt* [Table 1]. Indeed, pollen specific reactivity, as measured by the cumulative wheal reaction to SPT with pollen extracts, and total IgE levels were higher in patients with double sensitization than in the whole population of grass pollen sensitized patients [Table 1].

**Table 1:** Characteristics of subgroups of italian children with seasonal allergic rhinoconjunctivitis (SAR), according to their sensitization profiles.

	Sensitized to grass pollen (n = 1120)		Sensitized to grass and *D.pt* (n = 638)		Sensitized to grass and *D.pt* tested for IgE to Der p 1, Der p 2, Der p 23 (n = 146)^a^	
Age (years) (mean, sd)	10,54	3,42	10,76	3,31	10,99	3,16
Males (n, %)	772	68,99	451	70,8	109	74,17
Familiar atopy (n,%)						
Mother	492	43,93	283	44,36	62	42,47
Father	440	39,29	252	39,5	58	39,73
Geographic Area (n,%)						
North	370	33,04	225	35,27	60	41,1
Center	532	47,5	259	40,6	59	40,41
South & Islands	218	19,46	154	24,14	27	18,49
Characteristic of SAR						
Age at onset SAR (ys) (mean, sd)	5,33	2,87	4,99	2,64	4,9	2,54
Duration od SAR (ys) (mean, sd)	5,31	3,31	5,77	3,35	6,09	3,39
Moderate/severe SAR (n, %)	583	52,05	343	53,76	75	51,37
Months with symptoms (mean, sd)	4,28	1,79	4,42	1,78	4,59	1,84
Pollen season months with symptoms (mean, sd)	3,91	1,43	4,03	1,4	4,13	1,43
Winter season months with symptoms (mean, sd)	0,37	0,84	0,39	0,85	0,47	0,89
Asthma (n, %)	416	37,14	264	41,38	66	45,21
Oral allergy syndrome (n, %)	0,23	0,43	0,25	0,43	0,29	0,45
Atopic reactivity (mean, sd)						
Overall SPT reactivity to pollen (mm) (mean, sd)	36,48	21,11	37,27	21,9	41,08	22,55
Levels of total IgE (kU/L) (g mean, sd)	386,08	2,97	524,81	2,69	602,56	2,69

a No significant differences (p 0.05) between the randomized population (n 146) and the original study population (n 638)

### IgE levels to allergen extracts, by molecular spreading to grass pollen

We wanted to ascertain whether the spreading of the IgE response to grass pollen molecules is associated with a general tendency to polysensitization. To this end, we examined the level of the sensitization to pollen and non-pollen allergen extracts among grass pollen sensitized children, stratified by the number of allergen molecules of *Phleum pratense* recognized by their serum IgE [Table 2]. Geometric mean levels of IgE antibodies to the majority of pollen extracts (olive, ragweed, mugwort, birch, plane) increased with the number of *Phleum pretense* molecules recognized by patient's IgE. On the contrary, the geometric mean levels of IgE antibodies to the extract of *Dermatophagoides pteronyssinus* were inversely associated to the number of *Phleum pratense* molecules recognized by the patient's IgE. Additionally, the geometric mean levels of serum IgE antibodies to Alternaria and to cat allergen extracts were not associated to the number of *Phleum pratense* molecules recognized by the patient's IgE.

**Table 2:** Total and serum IgE levels to allergen extracts, by number of Phleum p. molecules recognized by IgE^a^.

	Number of Phleum pratense molecules recognized by IgE																
	1		2		3		4		5		6		7		8		p
	g mean	sd	g mean	sd	g mean	sd	g mean	sd	g mean	sd	g mean	sd	g mean	sd	g mean	sd	
Total IgE	200,68	2,81	325,23	3,23	367,10	2,94	350,45	2,81	407,04	2,51	562,58	2,43	720,43	2,57	2345,01	3,06	<0.001
sIgE to Phleum p.	1,01	1,94	5,38	2,96	10,42	3,22	21,80	3,18	58,52	3,20	107,64	3,11	163,74	2,96	277,17	5,61	<0.001
sIgE to Cynodon d.	0,94	2,57	2,69	3,09	5,77	4,27	4,95	4,39	10,46	3,39	18,71	3,05	35,88	2,88	29,49	3,08	<0.001
sIgE to Cupressus a.	3,74	3,60	3,63	3,61	4,97	3,65	3,51	3,51	4,41	4,02	4,33	4,42	4,73	3,94	25,77	5,43	0,093
sIgE to Parietaria j.	8,60	6,03	11,63	5,08	8,86	5,38	13,21	6,08	11,50	6,67	8,86	4,98	7,16	3,91	69,36	3,92	0,888
sIgE to Olea e.	2,44	4,02	3,99	3,80	4,37	4,80	5,05	3,48	5,43	3,45	7,83	2,96	13,92	2,83	40,54	4,00	<0.001
sIgE to Betula v.	3,25	4,69	4,25	4,49	9,59	5,20	5,89	6,51	7,48	5,13	9,32	4,21	15,15	3,78	100,79	4,52	<0.001
sIgE to Ambrosia a.	1,65	3,67	2,96	3,19	3,31	3,91	3,17	3,75	4,23	3,84	7,17	3,51	10,57	3,01	65,26	3,67	<0.001
sIgE to Artemisia v.	1,65	3,14	3,85	3,68	3,07	3,33	2,67	4,69	3,63	3,32	4,31	3,42	6,36	2,83	29,89	5,00	0,001
sIgE to Platanus a.	1,54	3,14	2,46	3,70	3,04	4,40	2,69	3,82	3,21	3,73	4,69	3,28	7,49	3,74	64,04	4,00	<0.001
sIgE to Dermatophagoides pt.^b^	11,15	8,74	19,12	8,71	14,49	8,10	14,13	6,70	5,39	6,55	4,16	6,03	3,54	5,36	3,43	8,45	<0.001
sIgE to alternaria	6,33	3,98	6,92	4,86	8,21	4,10	7,54	4,41	6,33	4,46	5,04	5,12	3,84	4,41	4,12	5,25	0,049
sIgE to cat	4,45	3,94	4,25	4,68	3,87	4,81	3,75	4,02	4,38	4,77	3,96	4,78	2,73	3,68	8,13	8,12	0,668

a IgE levels >0.35

b The cut off for specific IgE POSITIVITY IS >0.35 KU/L.

### Inverse association between the molecular spreading of the IgE reponse to D.pteronyssinus and grass pollen

**Fig. 2:**
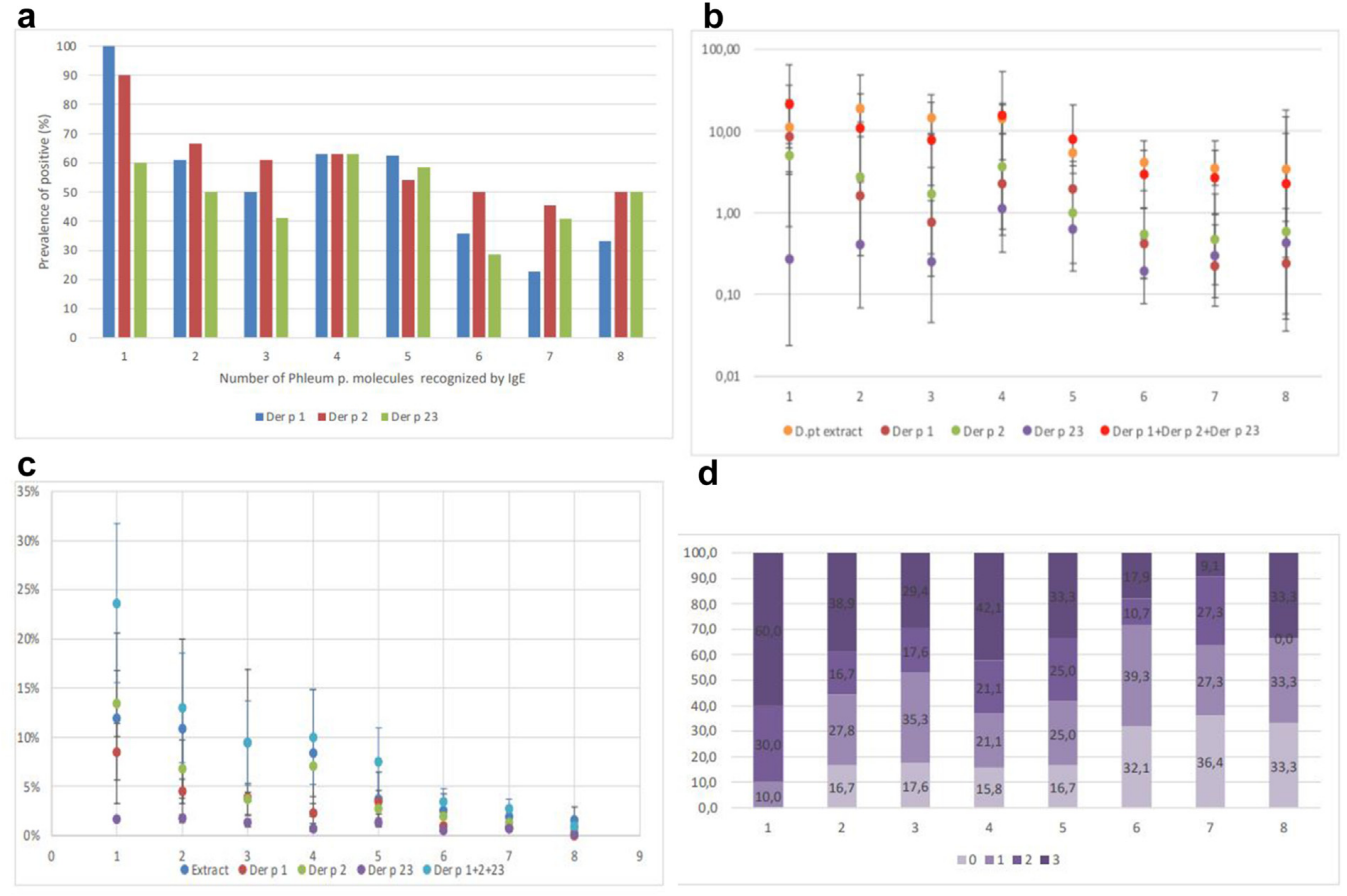
**–** IgE sensitization to Dermatophagoides pteronyssinnus and its major allergenic molecules, by number of Phleum pratense molecules recognized by IgE. For each subgroup and *D.pt* molecule, we calculated the percentage of positive outcome [Fig. 2a], geometric mean level [Fig. 2b], and the average specific activity of the serum IgE antibodies to Der p 1, Der p 2 and Der p 23 [Fig. 2c], the number of Dermatophagoides pteronyssinnus molecules recognized by sIgE, according to Phleum pratense ones [Fig. 2d]. The cumulative level of the IgE to these 3 antibodies [Fig. 2b] and the distribution of a molecular spreading index based on these 3 molecules only [Fig. 2a] are also shown. ***D.pt*:** Dermatophagoides pteronyssinus, **Phl p1**: Phleum pretense, **IgE:** Immunoglobulin IgE, **Der p1, p2, p23:** Dermatophagoides pteronyssinus.

To get further insights on the inverse association between IgE molecular spreading to grass pollen and IgE levels to *D.pt*, we measured IgE to rDer p 1, rDer p 2, rDer p 23 in the sera of a balanced subset of patients sensitized to both grass pollen and *D.pt*. To this end, we examined a total of 146 patients with sensitization to varied ammounts of grass pollen molecules: 6 with IgE to all 8 allergenic molecules of *Phleum pratense* and 140 sera randomly selected with IgE to 1, 2, 3, 4, 5, 6, and 7 allergenic molecules of *Phleum pratense*. For each subgroup and *D.pt* molecule, we calculated the percentage of positive outcome [Fig. 2a], geometric mean level [Fig. 2b], and the average specific activity of the serum IgE antibodies to Der p 1, Der p 2 and Der p 23 [Fig. 2c]. We also calculated the geometric mean value of the cumulative level of the IgE to these 3 antibodies [Fig. 2b] and the distribution of a molecular spreading index based on these 3 molecules only [Fig. 2d]. The results show that, independently from the methodology used to calculate the level of IgE sensitization to *D.pt* allergens, this level declined with the increasing number of *Phleum pratense* molecules recognized by the patient's serum IgE. Hence, a broader molecular spreading to grass pollen was related to a reduced molecular spreading to *Dermatophagoides pteronyssinus*.

### Univariate and multivariate analysis of the inverse association between IgE antibody levels to *D. pteronyssinus* and molecular spreading to grass pollen

The observed inverse association between grass and mite IgE response might have been spurious. Therefore, we performed a univariate and a multivariate linear analysis to test whether a series of potential confounders may explain the results, generating the association. In an univariate analysis, a younger age, living in Southern Italy (instead of North), and having symptoms of asthma outside the pollen season show significant association with the levels of specific IgE to *D.pt* [Table 3a]. However, after adjusting this analysis for the number of *Phleum Pratense* molecules recognized by IgE, only the presence of symptoms of asthma outside the pollen season and living region remain significantly associated with the levels of IgE to *D.pt* extract. In a multivariate linear model including all factors analyzed at univariable level, and total IgE as potential confounders, the number of grass pollen molecules recognized by IgE was still inversely associated to the level of IgE antibodies to *D.pt* extracts [Table 3b]. Interestingly, this analysis has shown that age does not play any confounding role in the observed inverse association between the IgE response to grass pollen and the IgE response to *Dermatophagoides pteronyssinus* [Table 3b].

**Table 3a:** Linear regression analysis of the levels of specific IgE to Dermatophagoides pteronyssinus in relation to the main characteristics of the study population and clinical outcomes.

	Univariate analysis		Adjusted for the number of *Phleum pratense* molecules recognized by IgE	
	Beta	p	Beta	p
Age	−0,07	0,04	−0,04	0,099
Gender	0,10	0,564	0,15	0,365
Geographic area		0,33		0,799
North	0			
Centre	0,094	0,614	0,055	0,754
South and Islands	0,52	0,013	1,36	0,504
Symotoms in May–June	0,07	0,677	0,02	0,89
Allergic rhinitis outside the pollen season	0,64	0,003	0,52	0,012
Asthma outside the pollen season	0,78	<0,001	0,72	<0.001
Asthma	0,21	0,203	0,27	0,081

The quantitative dependent variable was sIgE to *Dermatophagoides pteronyssinus.* Beta represents the estimated coefficient of regression for each variable of the model and p the relative p-value

**Table 3b:** Multivariable linear regression to test the effect of the number of Phleum pratense molecules recognized by IgE (>0,35 kUA/L) on the levels of specific IgE to *Dermatophagoides pteronyssinus*, adjusted for significant factors identified through univariate analysis.

	Multivariable linear regression	
	Beta	p
*Phleum pratense* molecules recognized by IgE (n)^a^	−0,32	<0.001
Total IgE	0,0003	<0.001
Age	−0,04	0,142
Gender	0,11	0,483
Allergic rhinitis outside pollen season	0,53	0,008
Asthma outside the pollen season	0,68	<0.001

The quantitative dependent variable was sIgE to *Dermatophagoides pteronyssinus*. Beta represents the estimated coefficient of regression for each variable of the model and p the relative p-value.

a cut off:≥ 0,35 kUA/L

## Discussion

### Key findings

In a large cross-sectional population of Italian children affected by grass pollen allergy (PAN PED cohort study), we found that a broader molecular response to *Phleum pratense* molecules is associated with: (1) higher total IgE levels; (2) pollen polysensitization and higher levels of IgE antibodies to pollen; (3) lower levels of IgE antibodies to the extract of *Dermatophagoides pteronyssinus*; and (4) lower degree of sensitization to rDer p 1, r Der p 2, and rDer p 23. Together, these data suggest that, at least in a consistent population subset, the propensity to produce a strong and molecularly diversified IgE to environmental allergens (atopy) is limited to pollen and that its expression is preventing or contrasting the development of a similar response to *Dermatophagoides pteronyssinus*.

Thus, having developed a complex and diverse IgE response for *Phleum pratense*, that is, the presence of positivity for multiple Phleum pratense molecules, appears to block or counteract a similar response toward a non-pollen allergen, such as *D.pt*.

### Molecular spreading to grass runs together with pollen polysensitization

The strong association in our Italian population of pollen-allergic children between molecular spreading of the IgE response to grass pollen with pollen polysensitization and higher total IgE serum levels is not surprising. In this same population, we have already shown that this molecular spreading, intra and inter allergen, is associated with parental atopy.^11^ The propensity to develop a molecular spreading for grass pollen was also associated with higher total IgE levels and sensitization to multiple pollen extracts (poly-sensitization).^1^ Similarly, studies within the Multicenter Allergy Study (MAS), a German birth cohort, demonstrated a strong influence of parental atopy in developing a broader response to grass pollen allergen molecules.^8^^,^^19^, ^20^, ^21^, ^22^

In the present study, we expand our previous findings, showing that patients with a stronger molecular spreading of IgE sensitization to grass pollen are also polysensitized to *Fagales*, *Oleaceae*, Pellitory, and *Cupressaceae* pollen. According to textbook knowledge, it can be easily concluded that a stronger atopic genetic predisposition facilitates intense and diversified IgE responses at a molecular level within the same pollen and also a broader poly-molecular IgE progression to different pollens.^2^, ^3^, ^4^, ^5^, ^6^^,^^7^

### Molecular spreading to grass and IgE sensitization to mites

The inverse association of a strong molecular spreading of the IgE response to grass pollen with mite-specific IgE levels and polymolecular sensitization to Der p 1, Der p 2, and Der p 23 is a surprising finding. One would expect that children with a stronger molecular spreading of the IgE response to grass pollen would have shown a higher prevalence and broader molecular response also to mite allergens. Moreover, the coexistence of mite and grass pollen allergy in children is very common in allergy practice 27, 28, 29, 30with AIT guidelines specifically created for this disease phenotype.^12^^,^^15^^,^^16^

In fact, several studies in the literature, focusing on grass and mite allergy, have increased our knowledge on this topic. In the large population subset of Manchester Asthma and Allergy Study (MAAS), Custovic et al studied the different nature of the longitudinal trajectories of the IgE response between grass pollen and mite associated with different clinical outcomes. Children with mite sensitization, independently of age, presented the highest risk of asthma, also associated with an early onset of grass pollen allergy. In contrast, a late onset of grass pollen allergy was associated with risk of AR.^27^

Later, among the same MAAS cohort, a detailed analysis of the IgE sensitization profiles and their evolution to multiple allergen components has been performed according to age. The authors described the IgE evolution patterns towards multiple allergens throughout childhood thanks to machine learning techniques, founding that an early onset of Phl p 1 and Phl p 5 in preschool age was associated with asthma, while their late onset in school-age children with AR. In addition, the statistically significant association of asthma at 16 years of age was the dust mite-sensitized cluster.^31^

These results were also confirmed, in another study conducted in the MAAS cohort, after a detailed analysis of the IgE sensitization profiles, applying machine learning techniques to component resolved diagnostics (CRD) and through a connectivity analysis of the interactions between the various allergenic components.

The authors identified a pairwise relationship between specific IgE and asthma risk, demonstrating that mite sensitization was statistically associated with an increased asthma risk.^32^

Another recent study investigated the role of CRD in identifying clinically relevant sensitizations in children at high risk of developing asthma. The authors found an increased asthma risk at 10 years of age in patients which showed higher levels of Derp1, Der p2, Der p23, Der f1, and Der f2, instead non-asthmatic children had higher serum concentration of Phl p4, Phl p5, Phl p6, and Phl p12.^33^

Thus, all the major studies reported, showed the very common occurrence of sensitization to both allergens, *Phleum pratense* and *D.pt*, focusing on the relative clinical implications of these sensitivities.

However, to date, no study has investigated the kind of relationship between these 2 different allergens and their relative molecular spread, either considering extracts or CRD.

### Interpretation

Different hypotheses may be raised to explain our outcome. Unfortunately, none of them can be excluded a priori, nor clarified within the context of the present study:

(1) **Selection bias -** The PAN-PED study recruited patients with a confirmed diagnosis of seasonal allergic rhinitis. The inverse association between mite and grass pollen sensitization may result from misdiagnosis. Mite allergic patients with a weak positive SPT reaction to grass pollen and a low, monomolecular IgE levels to Phl p 1, may have been misdiagnosed and recruited as grass pollen allergic children. The mix of 2 groups of patients, primarily sensitized to grass pollen and primarily sensitized to mites, may have artificially generated the observed inverse associations. A not significant inverse association was observed between IgE sensitization to grass and IgE sensitization to *Alternaria alternata* and cat. However the number of patients with *Alternaria alternata* and cat allergy is too low to extabilish a possible significance and we cannot exclude that it may be a general phenomenon rather than a specific allergenic phenomenon for mite.
(2) **Exposure bias –** Parents of children with pollen allergies might reduce their children's exposure to indoor allergens, particularly mites, as part of their child's allergy prevention plan. Unfortunately, there is no data supporting the concept that environmental dust mite prevention blocks the development of allergic sensitization to it. Instead, it was observed a beneficial effect of dust mite prevention is reducing exacerbations of allergic symptoms, particularly asthma, and relative hospitalizations.^34^, ^35^, ^36^, ^37^
(3) **Site-limited pre-priming of the IgE response to pollen –** The impact on patient's mucosa is more similar in terms of time frame (daytime) and sites (conjunctiva, turbinates) among different pollen types than among pollen versus mite faecal pellets or bodies. The Th2-driven, eosinophilic inflammation produced by grass pollen might preferentially stimulate the onset of further pollen sensitization rather than mites or other indoor allergens.

The intrinsic characteristics of antigens are crucial in determining allergenicity, triggering reactions, and activating inflammatory responses in the respiratory airways, depending on their different molecular structures.^38^ Der p1, a serine protease and Der p 2, a non-proteolytic allergen of D.pteronyssinus, can enhance epithelial permeability stimulating respiratory epithelial cells and activating a pro-inflammatory response. Conversely, Phl p1 and Phl p5 can stimulate upper airway cells and promote allergens intake, being a beta-expansin and a ribonuclease.^39^, ^40^, ^41^ However, by following their different intrinsic structures, the association between allergy to grass pollens and other pollens was observed more often than to grass pollen and indoor allergens, such as dust mites.^40^, ^41^, ^42^

(4) **Higher specific activity favours the first sensitizing allergen –** It has been shown that activation of degranulation on Mast-cells and Basophils is highly influenced by IgE-specific activity.^43^^,^^44^ Longitudinal studies in the MAS birth cohort have also shown that the highest peaks of IgE levels are achieved by very early allergen responses, while those starting later in life are also weaker.^21^^,^^22^ Therefore, those IgE responses starting earlier and progressing wider in childhood also reduce the possibility that a later response to mite allergens develops strong and molecularly widespread.

We may hypothesize that the first allergen inducing an allergic, immunologic response dominates the future evolution of the allergic sensitization picture both in terms of clinical response and serologic IgE evolution. In other words, if the allergic response begins with a specific allergen, the clinical picture will be dominated by this allergen with increased symptoms after contact with it. All the other allergens to which sensitization will subsequently develop may have a different clinical relevance. Poly-sensitization does not necessarily indicate the presence of more severe disease. This concept is also true regarding serological responses. IgE levels follow the order of sensitization's onset: higher for the first allergen to appear and lower for subsequent allergens according to the order of the appearance of allergens during sensitization. This happens for both extracts and molecules. However, this hypothesis deserves further studies with a longitudinal approach.

### Limitations

We should acknowledge some limitations in our observational study. The first is the possibility of a selection bias or reverse causation and exposure bias, as discussed above. In addition, our population sample is limited to a Mediterranean country, and its conclusions cannot be extrapolated to other world areas (eg, central or northern Europe), characterized by a different climate and much lower diversity of pollen exposure. Third, the cross-sectional nature of our study prevented us from reaching clear-cut conclusions, while a longitudinal study design of a birth cohort would have been a more powerful study design to answer our study question.

However, as today we are trying to analyze this hypothesis in a birth cohort study to determine whether the inverse association persists over time.

### Perspectives

In conclusion, we found an inverse association between the molecular spreading of the IgE response to grass pollen and that to Dermatophagoides pteronyssinus in the PAN-PED cohort study population. Although we could not exclude potential bias, our findings raise questions concerning the influence of the earliest IgE responses and the eosinophilic inflammation they provoke on the natural history and biological development of the IgE responses following the first one. We are currently investigating this hypothesis in a birth cohort study. Such a study may also test whether the inverse association we have observed is not only limited to molecularly diversified patterns of IgE sensitization to pollen or mites, but ecompasses also a broader tendency to develop SAR or Asthma.

## Funding

None.

## Availability of data and materials

The raw data supporting the conclusions of this article will be made available by the authors, without undue reservation.

## Author contribution

GB and FC contributed equally to this work. PMM contributed to the conception and design of the study. GB, FC, EP, ST, RB, CC, AC, RC, LC, PC, GD, MMDG, IDI, ADRB, SD, MG, AG,VM, IS,EV, GR,GR,AMZ contributed to the acquisition of data. VP performed the statistical analysis of data under the supervision of PMM. GB, FC, VP, EK, and PMM contributed to the interpretation of the results and in drafting the manuscript. All authors revised and approved the final version of the manuscript.

## Ethics approval

The PAN-PED FU study design and procedures have been approved by the ethics committee of S. Orsola-Malpighi Hospital of Bologna (103/2009/O/Oss, EM 178/2014/O and 210/2015/U) and of all the participating centers.

## Authors’ consent for publication

All the authors gave their consent for publication.

## Declaration of competing interest

Dr. P. Matricardi reports grants and personal fees from Euroimmun AG; grants and personal fees from Thermo Fisher Scientific, personal fees from Hycor Biomedical Inc, outside the submitted work. Dr S. Tripodi is cofounder of TPS Production. All other authors declared no conflicts of interest.

## References

[1] Brożek J.L., Bousquet J, Agache I. (2017). Allergic rhinitis and its impact on asthma (ARIA) guidelines-2016 revision. J Allergy Clin Immunol.

[2] De Vittori V., Pacilio A, Indinnimeo L (mag. 2019). When asthma and rhinitis coexist, could rhinitis reduce asthma control in children?. Allergy Asthma Proc.

[3] Cruz A.A., Popov T, Pawankar R (2007). Common characteristics of upper and lower airways in rhinitis and asthma: ARIA update, in collaboration with GA(2)LEN. Allergy.

[4] van Vliet D., Essers BA, Winkens B (2020). Longitudinal relationships between asthma-specific quality of life and asthma control in children; the influence of chronic rhinitis. J Clin Med.

[5] Schuler C.F., Montejo e J.M. (2019). Allergic rhinitis in children and adolescents. Pediatr Clin.

[6] Brindisi G., De Vittori V, De Nola R (mar. 2021). The role of nasal nitric oxide and anterior active rhinomanometry in the diagnosis of allergic rhinitis and asthma: a message for pediatric clinical practice. J Asthma Allergy.

[7] Dondi A., Tripodi S, Panetta V (2013). Pollen-induced allergic rhinitis in 1360 Italian children: comorbidities and determinants of severity. Pediatr Allergy Immunol.

[8] Hatzler L., Panetta V, Lau S (2012). Molecular spreading and predictive value of preclinical IgE response to Phleum pratense in children with hay fever. J Allergy Clin Immunol.

[9] Asher M.I., Montefort S, Björkstén B (2006). Worldwide time trends in the prevalence of symptoms of asthma, allergic rhinoconjunctivitis, and eczema in childhood: ISAAC Phases One and Three repeat multicountry cross-sectional surveys. Lancet Lond. Engl..

[10] Scala E., Villalta D, Uasuf CG (2018). An atlas of IgE sensitization patterns in different Italian areas. A multicenter, cross-sectional study. Eur. Ann. Allergy Clin. Immunol..

[11] Cipriani F., Tripodi S, Panetta V (2019). Early molecular biomarkers predicting the evolution of allergic rhinitis and its comorbidities: a longitudinal multicenter study of a patient cohort. Pediatr. Allergy Immunol. Off. Publ. Eur. Soc. Pediatr. Allergy Immunol..

[12] Matricardi P.M., Dramburg S., Potapova E., Skevaki C., Renz e H. (mar. 2019). Molecular diagnosis for allergen immunotherapy. J Allergy Clin Immunol.

[13] Dramburg S., Matricardi e P.M. (2019). Molecular diagnosis of allergy: the pediatric perspective. Front. Pediatr..

[14] Matricardi P.M. (ago. 2013). Molecular profile clustering of IgE responses and potential implications for specific immunotherapy. Curr Opin Allergy Clin Immunol.

[15] Matricardi P.M., Kleine-Tebbe J, Hoffmann HJ (2016). EAACI molecular allergology user’s guide. Pediatr. Allergy Immunol. Off. Publ. Eur. Soc. Pediatr. Allergy Immunol..

[16] Alvaro-Lozano M., Akdis CA, Akdis M (2020). EAACI allergen immunotherapy user’s guide. Pediatr. Allergy Immunol. Off. Publ. Eur. Soc. Pediatr. Allergy Immunol..

[17] Schwarz A., Panetta V, Cappella A (nov. 2016). IgG and IgG4 to 91 allergenic molecules in early childhood by route of exposure and current and future IgE sensitization: results from the Multicentre Allergy Study birth cohort. J Allergy Clin Immunol.

[18] Tripodi S., Frediani T., Lucarelli S. (2012). Molecular profiles of IgE to Phleum pratense in children with grass pollen allergy: implications for specific immunotherapy. J Allergy Clin Immunol.

[19] Hatzler L., Panetta V, Illi S (giu. 2014). Parental hay fever reinforces IgE to pollen as pre-clinical biomarker of hay fever in childhood. Pediatr. Allergy Immunol. Off. Publ. Eur. Soc. Pediatr. Allergy Immunol..

[20] Bergmann R.L., Bergmann KE, Lau-Schadensdorf S (1994). Atopic diseases in infancy. The German multicenter atopy study (MAS-90). Pediatr. Allergy Immunol. Off. Publ. Eur. Soc. Pediatr. Allergy Immunol..

[21] Matricardi P.M., Bockelbrink A, Keil T (2009). Dynamic evolution of serum immunoglobulin E to airborne allergens throughout childhood: results from the Multi-Centre Allergy Study birth cohort. Clin. Exp. Allergy J. Br. Soc. Allergy Clin. Immunol..

[22] Posa D., Perna S, Resch Y (2017). Evolution and predictive value of IgE responses toward a comprehensive panel of house dust mite allergens during the first 2 decades of life. J Allergy Clin Immunol.

[23] Peroni D.G., Piacentini G.L., Bodini A., Rigotti E., Pigozzi R., Boner e A.L. (mar. 2008). Prevalence and risk factors for atopic dermatitis in preschool children. Br J Dermatol.

[24] Kang H., Yu J., Yoo Y., Kim D.K., Koh e Y.Y. (ago. 2005). Coincidence of atopy profile in terms of monosensitization and polysensitization in children and their parents. Allergy.

[25] Silvestri M., Oddera S., Crimi P., Rossi e G.A. (1997). Frequency and specific sensitization to inhalant allergens within nuclear families of children with asthma and/or rhinitis. Ann. Allergy Asthma Immunol. Off. Publ. Am. Coll. Allergy Asthma Immunol..

[26] Lau S., Matricardi P.M., Wahn U., Lee Y.A., Keil e T. (gen. 2019). Allergy and atopy from infancy to adulthood: messages from the German birth cohort MAS. Ann. Allergy Asthma Immunol. Off. Publ. Am. Coll. Allergy Asthma Immunol..

[27] Custovic A., Sonntag H.-J., Buchan I.E., Belgrave D., Simpson A., Prosperi e M.C. F. (2015). Evolution pathways of IgE responses to grass and mite allergens throughout childhood. J Allergy Clin Immunol.

[28] Scala E., Alessandri C, Bernardi ML (giu. 2010). Cross-sectional survey on immunoglobulin E reactivity in 23,077 subjects using an allergenic molecule-based microarray detection system. Clin. Exp. Allergy J. Br. Soc. Allergy Clin. Immunol..

[29] López Freire S., Méndez Brea P., González Fernández T., Gude F., Vidal e C. (2021). Interference of Dermatophagoides pteronyssinus sensitization in grass pollen allergy. Eur. Ann. Allergy Clin. Immunol..

[30] Kiewiet M.B.G., Lupinek C, Vrtala S (feb. 2023). A molecular sensitization map of European children reveals exposome- and climate-dependent sensitization profiles. Allergy.

[31] Howard R., Belgrave D., Papastamoulis P., Simpson A., Rattray M., Custovic e A. (ott. 2018). Evolution of IgE responses to multiple allergen components throughout childhood. J Allergy Clin Immunol.

[32] Fontanella S., Frainay C., Murray C.S., Simpson A., Custovic e A. (2018). Machine learning to identify pairwise interactions between specific IgE antibodies and their association with asthma: a cross-sectional analysis within a population-based birth cohort. PLoS Med.

[33] Farraia M., Mendes F.C., Sokhatska O. (2023 Jun). Sensitization trajectories to multiple allergen components in a population-based birth-cohort. Pediatr Allergy Immunol.

[34] Murray C.S., Foden P, Sumner H (2017 Jul 15). A randomized trial of mite-impermeable bedcovers. Am J Respir Crit Care Med.

[35] Woodcock A., Lowe LA, Murray CS (2004 Aug 15). Early life environmental control: effect on symptoms, sensitization, and lung function at age 3 years. Am J Respir Crit Care Med.

[36] Simpson A., Simpson B, Custovic A, Craven M, Woodcock A (2003 Sep). Stringent environmental control in pregnancy and early life: the long-term effects on mite, cat and dog allergen. Clin Exp Allergy.

[37] Gøtzsche P.C., Johansen HK, Burr ML, Hammarquist C (2001). House dust mite control measures for asthma. Cochrane Database Syst Rev.

[38] Traidl-Hoffmann C., Jakob T, Behrendt H (mar. 2009). Determinants of allergenicity. J Allergy Clin Immunol.

[39] Osterlund C., Grönlund H, Gafvelin G, Bucht A (2011). Non-proteolytic aeroallergens from mites, cat and dog exert adjuvant-like activation of bronchial epithelial cells. Int Arch Allergy Immunol.

[40] Röschmann K., Farhat K, König P, Suck R, Ulmer AJ, Petersen A (2009 Sep). Timothy grass pollen major allergen Phl p 1 activates respiratory epithelial cells by a non-protease mechanism. Clin Exp Allergy.

[41] Göbl C., Focke-Tejkl M, Najafi N (2017 Oct). Flexible IgE epitope-containing domains of Phl p 5 cause high allergenic activity. J Allergy Clin Immunol.

[42] Ricci G., Righetti F, Menna G, Bellini F, Miniaci A, Masi M (2005 Jun). Relationship between Bet v 1 and Bet v 2 specific IgE and food allergy in children with grass pollen respiratory allergy. Mol Immunol.

[43] Christensen L.H., Holm J, Lund G, Riise E, Lund K (2008). Several distinct properties of the IgE repertoire determine effector cell degranulation in response to allergen challenge. J Allergy Clin Immunol.

[44] Hazebrouck S., Canon N, Dreskin SC (feb. 2022). The effector function of allergens. Front. Allergy.

